# Effects of Oral Glutamine Supplementation on Early Postnatal Muscle Morphology in Low and Normal Birth Weight Piglets

**DOI:** 10.3390/ani10111976

**Published:** 2020-10-28

**Authors:** Yaolu Zhao, Elke Albrecht, Miriama Sciascia, Zeyang Li, Solvig Görs, Johannes Schregel, Cornelia C. Metges, Steffen Maak

**Affiliations:** 1 Institute of Muscle Biology and Growth, Leibniz Institute for Farm Animal Biology (FBN), 18196 Dummerstorf, Germany; zhao@fbn-dummerstorf.de (Y.Z.); maak@fbn-dummerstorf.de (S.M.); 2 Institute of Nutritional Physiology “Oskar Kellner”, Leibniz Institute for Farm Animal Biology (FBN), 18196 Dummerstorf, Germany; sciascia@fbn-dummerstorf.de (M.S.); li@fbn-dummerstorf.de (Z.L.); goers@fbn-dummerstorf.de (S.G.); schregel@fbn-dummerstorf.de (J.S.); metges@fbn-dummerstorf.de (C.C.M.)

**Keywords:** amino acids, glutamine supplementation, intramuscular lipid deposition, low birth weight, myosin heavy chain isoforms, piglets, skeletal muscle morphology

## Abstract

**Simple Summary:**

The lower survival rate and diminished overall growth and performance of low birth weight piglets is an economic and ethical issue in pig production. Adapted nutrition can help to compensate growth retardation, in particular, if nutrients in milk are not sufficient for newborn piglets. This study investigated the effects of oral glutamine supplementation during the first days after birth on skeletal muscle morphology of piglets with low or normal birth weight. The results indicated that glutamine supplementation changed the intramuscular amino acid concentration in the short term and influenced the muscle fiber size and abundance of myosin heavy chain isoforms within longissimus and semitendinosus muscle in neonatal piglets. Furthermore, differences in muscle fiber size and intramuscular lipid deposition were observed between low and normal birth weight piglets that disappeared in older piglets, independent of supplementation. The results indicated a delayed muscle development in low birth weight piglets. This delay can partly be compensated during growth by adequate nutrition.

**Abstract:**

Adapted nutrition can improve the growth of low birth weight (LBW) piglets. Since maternal milk is thought to provide insufficient glutamine (Gln) for LBW piglets, the current study investigated the influence of Gln supplementation during the early suckling period on development and lipid deposition in skeletal muscle. The weight differences between LBW and normal birth weight (NBW) littermates persisted from birth to slaughter (*p* < 0.001). However, intramuscular Gln and Ala concentrations were altered in piglets according to the supplementation (*p* < 0.01). There were larger muscle fibers (*p* = 0.048) in Gln-supplemented piglets. Capillarization or nuclei number per muscle fiber was not influenced by birth weight (BiW) or Gln supplementation. Abundance of myosin heavy chain (MYH) isoforms was slightly altered by Gln supplementation. LBW piglets had more lipid droplets than NBW piglets at day 5 of life in both muscles (*p* < 0.01). The differences decreased with age. Adipocyte development increased with age, but was not influenced by BiW or supplementation. The results indicate that BiW differences were accompanied by differences in lipid deposition and muscle fiber structure, suggesting a delayed development in LBW piglets. Supplementation with Gln may support piglets to overcome those disadvantages.

## 1. Introduction

The increased demand for pork has led to selection for larger litter sizes in pig production which has resulted in increased variation of piglet birth weight (BiW) within a litter [1]. About 15–20% of piglets in one litter are affected by low birth weight (LBW) or intrauterine growth restriction (IUGR) caused by inadequate maternal nutrition and insufficient placental functions [2,3]. These animals exhibit lower survival rates, slower postnatal growth and faster deposition of body fat in comparison with normal or heavy birth weight piglets [4]. Furthermore, LBW piglets have lower insulin sensitivity [5] and changes in liver lipid and amino acid (AA) metabolism [6]. Thus, IUGR piglets or LBW piglets have lower muscle growth and increased fat deposition [7], resulting in higher carcass lipid content and diminished meat quality at slaughter [2]. However, adapted nutrition can be used to help LBW piglets partially compensate for the associated negative impacts on growth and developmental retardation. It has previously been reported that milk-fed piglets benefit from additional milk intake, while milk has traditionally been thought to provide adequate amounts of all amino acids to neonates [8]. However, studies suggest that dietary glutamine (Gln) provided by milk may be inadequate for protein synthesis of piglets [9] and additional Gln requirements were postulated, especially for LBW piglets [10].

Glutamine, as the most abundant free AA in the body, has its primary source in skeletal muscle and plays an important part in the body growth [11]. It is an energy substrate for dividing cells, supplying intracellular protein turnover with ATP [11]. Furthermore, Gln provides nitrogen and carbon skeletons [12] for endogenous arginine synthesis, stimulates mammalian target of rapamycin (mTOR) pathway [13], takes part in the synthesis of glutathione [14], acts as a precursor for the synthesis of purine and pyrimidine nucleotides [11], is involved in maintaining intestinal barrier integrity and function and promotes cell proliferation [15]. Overall, Gln is of great importance in muscle tissue growth and a lack of Gln is possibly detrimental to normal development during the early postnatal period of piglets [10]. Studies have shown that it is possible to increase the muscle fiber number in piglets during the first days of life by nutrition [16,17]. Thus, stimulation of muscle fiber growth could improve development of skeletal muscle tissue und overall growth of piglets.

The high fat intake via maternal milk diet before weaning supplies the necessary energy for the build-up of body fat that occurs during this period in piglets. Piglets have almost no body fat at the beginning of their life compared to other mammals, e.g., human and guinea pig neonates, who have approximately 10% body fat at birth [18,19]. Until weaning, fat deposition increases tremendously because of the enhanced ability of piglets to assimilate and transfer dietary fat into fat cells [20,21]. After weaning, lipogenic activity is increased. As the growth rate in neonatal piglets is higher compared to other stages of life [22], sufficient nutrient supply is the most important issue during this period. A better understanding and improvement of LBW piglet management, in particular nutritional strategy, are therefore required for both animal welfare and productivity. As BiW is highly related to porcine meat quality, this study investigated the early development of the Musculus longissimus (MLD) and Musculus semitendinosus (MST), which are both well-established and common models in meat quality determination [23].

We hypothesized that oral Gln supplementation during the early postnatal phase can improve muscle development and body composition, particularly in LBW piglets. The current study focused on both the effects of BiW on the development of muscle morphology, including muscle fibers, intramuscular lipids, adipocytes and capillarization during the early postnatal period and on their modulation with Gln supplementation.

## 2. Materials and Methods

### 2.1. Animals and Sampling

All experimental procedures followed the Directive 2010/63/EU (European Convention for the Protection of Vertebrate Animals used for Experimental and Other Scientific Purposes) and were approved by the responsible State Office for Agriculture, Food Safety and Fishing Mecklenburg-Western Pomerania, Germany (permission No. 7221.3-1-026/16). Four groups were generated, consisting of 144 male German Landrace piglets, 72 LBW (BiW: 0.8 to 1.2 kg) and 72 normal birth weight (NBW) littermates (BiW: 1.4 to 1.8 kg). The LBW piglets were defined as piglets with weight below the lowest BiW quartile of the Leibniz Institute for Farm Animal Biology pig facility (FBN, Dummerstorf, Germany) [24], from where the piglets were sourced. Only six piglets were classified as IUGR according to Amdi et al. [25]. The piglets were supplemented with either 1 g Gln/kg body weight between day 1 and 12 after birth (the day of birth was considered as day 0), or an isonitrogenous amount of alanine (Ala, 1.22 g/kg body weight). The four groups were named LBW-GLN, NBW-GLN, LBW-ALA and NBW-ALA. The Gln dose from the current study was chosen based on the study published by Haynes et al. [26], who showed this dosage has beneficial effects in piglets aged 7–14 days. The supplemented Gln and Ala were prepared from fresh powder (AppliChem, Darmstadt, Germany and Ajinomoto, Tokyo, Japan) mixed with 2 mL water and were syringe-fed to the piglets three times every day at 07:00, 12:00 and 17:00. Free access to water and feed was provided to the sows during gestation and the lactation period. Twelve piglets per group were slaughtered at postnatal days 5, 12 and 26 (dpn—days post natum), respectively. All piglets were fed a dosage of 3 to 20 mL milk (depending on age of piglets; 150 g/ L water dissolved at 45 °C, Neopigg Rescuemilk 2.0, Provimi, Rotterdam, The Netherlands) and 33% of Gln and Ala of their daily intake, respectively, 2 h before slaughter.

Piglets slaughtered at 26 dpn were nursed by their respective dams and supplemented with either Gln or Ala until 12 dpn. From 14 dpn, the piglets had access to creep feed. Muscle tissue of the MLD and MST was taken immediately after slaughter separately for histology and for protein isolation, and snap frozen in liquid nitrogen. The tissue samples were stored at −80 °C for further experiments. Whole muscle cross sections from both muscles were cut about 1 cm thick and stored at −20 °C for determination of muscle cross sectional area.

### 2.2. Measurement of Free Amino Acid Concentrations within M. Longissimus

Muscle tissue of 30 mg fresh weight from each piglet was thawed and homogenized on ice with 300 μL lysis buffer according to the method described by Nebendahl et al. [27] with slight modifications, i.e., Tris was replaced by 10 mM Hepes, Igepal by Tween 20 and no Phospho-Stop solution was used. Samples were sonicated using an ultrasonic tip (Sonotrode MS 0.5, Carl Roth, Stuttgart, Germany) with 25 pulses for 0.5 s each and 80% amplitude, then centrifuged with 3000× *g* for 10 min at 4 °C, and stored at −20 °C until further analysis. The supernatant, tenfold diluted by ultra-pure water, was used for HPLC analysis as described in detail by Kuhla et al. [28], but with a HyperClone column (Phenomenex, Aschaffenburg, Germany). All free amino acid (AA) concentrations are presented as µmol per 1 kg fresh weight (µmol/kg).

### 2.3. Histology and Histochemistry

Serial sections of frozen MLD and MST were cut 10 µm thick with a cryostat microtome (CM3050 S, Leica, Bensheim, Germany). Standard protocols were used to stain intramuscular lipids with Oil Red O (Chroma Gesellschaft, Münster, Germany), to stain capillaries with alkaline phosphatase/eosin and nuclei with hematoxylin and eosin (H/E, hematoxylin: Dako, Glostrup, Denmark; eosin: Chroma Gesellschaft, Münster, Germany). An Olympus BX43 microscope (Olympus, Hamburg, Germany) equipped with a UC30 color camera and Cell^D imaging software (OSIS, Münster, Germany) was used to analyze intramyocellular lipid droplets, intramuscular adipocyte size, muscle fiber size, capillary size and density, as well as nuclei number. Respective self-made macro programs within the Cell^D software (OSIS, Münster, Germany) were used as described by Zitnan [29] and Dahl [30]. For analysis of intramyocellular lipid droplets, at least five randomly selected regions were analyzed for each piglet, accounting for a total area of about 0.75 mm^2^. To determine rare intramuscular adipocytes, a larger total area of about 1 cm^2^ was analyzed for each piglet. Capillary density and nuclei number were measured in three randomly selected regions with a size of about 1 mm^2^ and 0.1 mm^2^, respectively. Total apparent muscle fiber number was calculated by multiplication of muscle fiber number per mm^2^ and mean cross sectional area of both sides of the muscle tissue slices. Pictures of muscle slices including a ruler were taken by a digital camera (Coolpix 8700, Nikon, Düsseldorf, Germany) and measured with the interactive measurement module of the Cell^D software.

### 2.4. Immunohistochemistry

Muscle sections of MLD were cut 12 µm thick using a cryostat microtome (CM3050 S, Leica, Bensheim, Germany) and stained with antibodies against four myosin heavy chain (MYH) isoforms, namely MYH1, 2, 4 and 7. Antibodies against MYH isoforms (antigen homology to porcine proteins >94%) were purchased from antibodies-online (Aachen, Germany; ABIN6570793, ABIN2916107, ABIN6263466, and ABIN3043105). In brief, muscle sections were fixed in 4% paraformaldehyde (Carl Roth, Karlsruhe, Germany) for 15 min and then washed 2× 5 min with phosphate-buffered saline (PBS) and permeabilized with 0.1% Triton X100 (Sigma-Aldrich, Munich, Germany) in PBS for 10 min. After a blocking step with 10% normal goat serum (NGS) in PBST for 15 min, slides were incubated with primary antibodies (MYH1, 2, 4 and 7 diluted at 1:100 in PBST with 2% NGS) for 2 h. Then, the slides were rinsed briefly and washed 3× in PBST for 10 min each. Secondary antibodies (1:1000, Alexa Fluor 488 goat anti-rabbit IgG for MYH1, 2 and 4, Alexa Fluor 594 goat anti-mouse for MYH7, Life Technologies, Darmstadt, Germany) were incubated for 45 min in the dark. After that, the slides were rinsed briefly and washed in PBS for 3× 10 min and the nuclei were stained with Hoechst 33258 (Sigma-Aldrich, Munich, Germany) for 5 min. Finally, the slides were washed and covered with ProLong Antifade (Thermo Fisher Scientific, Schwerte, Germany) and respective coverslips (Carl Roth, Karlsruhe, Germany). All incubations were done at room temperature (RT) in a humidity chamber. Negative controls, omitting the primary antibody, were generated to detect unspecific bindings of the secondary antibodies. No unspecific binding was detected. A Cell^F image analysis system (OSIS, Münster, Germany) equipped with a Nikon Microphot SA fluorescence microscope (Nikon, Düsseldorf, Germany) and a CC-12 color camera was used to localize myosin isoforms in muscle cross sections.

### 2.5. Western Blotting

Proteins were extracted from the MLD and MST with CelLytic MT lysis reagent (Sigma-Aldrich, Munich, Germany) and protease inhibitor as described in detail by Liu et al. [31]. Protein abundance of MYH isoforms was determined with a Jess Simple Western system (ProteinSimple, San Jose, CA, USA) according to the manufacturer’s instructions. Briefly, a mixture of 2 µL (1 µg) protein sample, 2 µL of 0.1× sample buffer and 1 µl of 5× Mastermix was incubated at 95 °C, briefly vortexed and centrifuged, and placed on ice before pipetting into the plate. Primary antibodies (MYH1, 4 at 1:40, MYH2 at 1:25, MYH7 at 1:100) and secondary antibodies (rabbit IR and mouse NIR at 1:20; Bio-Techne, Wiesbaden, Germany) were diluted. Plates were loaded with protein mixture, diluted antibodies, buffers and protein ladder, as well as normalization reagent as loading control (ProteinSimple, San Jose, CA, USA). Then the procedure was performed with standard incubation times and signals were recorded in the NIR, IR and PN channel for the targets and total protein, respectively. The results were analyzed with the Compass for SW software (ProteinSimple, San Jose, CA, USA). Protein abundance was calculated by dividing the band volume of the target protein by the volume of total protein and given as relative protein abundance.

### 2.6. Statistical Analysis

All data were analyzed with SAS statistical software (Version 9.4, SAS Inst., Cary, NC, USA) by the analysis of variance (ANOVA) model using the MIXED procedure with BiW (LBW, NBW), supplementation (ALA, GLN), age (5, 12, 26) and respective interactions as fixed factors and sow as a random factor. Least square means (LSmeans) and standard errors (SE) were calculated for each fixed effect and pairwise differences were tested by the Tukey–Kramer test. The SLICE statement of the MIXED procedure was used for partitioned analyses of the LSmeans for the interaction between BiW and supplementation within ages. Differences were considered significant if Tukey–Kramer adjusted *p* ≤ 0.05 and a trend if 0.1 > *p* > 0.05. Pearson correlation coefficients were calculated using the CORR procedure of SAS.

## 3. Results

### 3.1. Slaughter Weights

**Figure 1:**
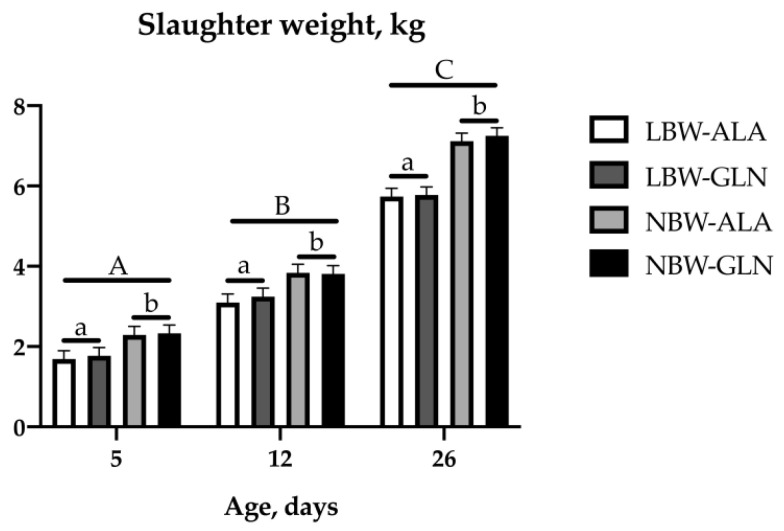
Slaughter weights of low birth weight (LBW) piglets and their normal birth weight (NBW) littermates supplemented with glutamine (GLN) or an isonitrogeneous amount of alanine (ALA) between 1 and 12 days post natum (dpn). Twelve piglets per group were each slaughtered at 5, 12, or 26 dpn; ^a,b^ indicate significant differences between LBW and NBW piglets at the same age; ^A–C^ indicate significant differences among ages (*p* ≤ 0.05, Tukey–Kramer test).

Low birth weight piglets were on average about 25% lighter at birth than their NBW littermates (Figure 1). The weight difference remained significant until slaughter (*p* < 0.001). There was no supplementation effect detected (*p* = 0.539). Birth weight and slaughter weight were highly correlated in the 5 dpn piglets (r = 0.898, *p* < 0.001) and moderately correlated in the 12 and 26 dpn piglets (r = 0.663 and 0.669, respectively, *p* < 0.001).

### 3.2. Concentrations of Free Amino Acids within M. Longissimus

The concentrations of free AAs and their metabolites in MLD were measured to determine whether the supplemented AA reached the muscle tissue and could have modulated the cellular development during the early postnatal phase. Concentrations of intramuscular AAs and their metabolites are presented in Table 1. As intended, the Gln concentration was higher in MLD of Gln-supplemented piglets at 5 dpn (*p* = 0.001) compared to Ala-supplemented piglets. However, within the LBW animals, the Gln concentration was not significantly higher in supplemented piglets due to the high individual variation. No difference in Gln concentration was observed at 12 dpn (*p* = 0.166) or 26 dpn (*p* = 0.373) between Gln- and Ala-supplemented animals. Similarly, the Ala concentration was higher in Ala-supplemented piglets at 5 dpn (*p* < 0.001) and at 12 dpn (*p* < 0.001), but this effect disappeared in piglets at 26 dpn (*p* = 0.730) compared to Gln supplementation. Concentrations of Gln and Ala metabolites were changed as well. Glutamic acid (Glu) tended to be lower at 12 dpn (*p* = 0.053), whereas citrulline (Cit) tended to be higher at 5 dpn (*p* = 0.069) in Gln-supplemented piglets. Moreover, BiW differences were found for aspartic acid (Asp, *p* = 0.022 at 5 dpn), ornithine (Orn, *p* = 0.034 at 12 dpn), proline (Pro, *p* = 0.003 at 5 dpn) and glycine (Gly, *p* = 0.032 at 5 dpn and *p* = 0.047 at 12 dpn) concentrations (Table 1). For indispensable AAs, concentration of leucine (Leu) tended to be higher at 5 dpn (*p* = 0.057) and was higher at 12 dpn (*p* = 0.001) in LBW piglets compared to NBW, while more isoleucine (Ile) and valine (Val) were measured in LBW piglets at 5 (*p* = 0.028, *p* = 0.003) and 12 dpn (*p* = 0.017, *p* < 0.001). The lysine (Lys) concentration was higher in LBW compared to NBW piglets at 5 (*p* = 0.043) and 26 dpn (*p* = 0.004). Furthermore, more Val was measured in Gln- than Ala-supplemented piglets at 12 dpn (*p* = 0.033). On the other hand, the concentration of carnosine (Car) was higher (*p* = 0.007), while that of anserine (Anser) tended to be higher (*p* = 0.06) in Gln-supplemented compared to Ala-supplemented piglets at 12 dpn. The higher concentration of beta-alanine (β-Ala) in LBW piglets at 5 dpn (*p* = 0.005) became a trend at 12 dpn (*p* = 0.053) and Anser concentration was lower in LBW piglets compared to NBW ones at 12 (*p* = 0.019) and 26 dpn (*p* = 0.001).

### 3.3. Muscle Morphology and Capillarization

Morphology and composition of two different muscles were investigated to determine whether the observed changes in AA concentrations and presumably higher availability of important AAs stimulated their development and growth. The LSmeans and SE for the BiW × Sup × Age interaction, BiW × Sup over ages and for Age over groups are shown in Table 2 and Table 3 for the MLD and MST, respectively. Muscle cross sectional area (CSA) reflected the differences in slaughter weights between LBW and NBW piglets. Overall, the CSA of both muscles was influenced by Age and BiW group but not by supplementation. Muscle CSA of MLD and slaughter weight correlated increasingly from r = 0.422 (*p* = 0.003) at 5 dpn to r = 0.702 (*p* < 0.001) at 12 dpn and r = 0.835 (*p* < 0.001) at 26 dpn. Similar values were recorded for the MST (r = 0.41, *p* = 0.004; r = 0.71, *p* < 0.001; r = 0.78, *p* < 0.001 for 5, 12 and 26 dpn, respectively).

Muscle fiber size (Table 2 and Table 3) was greater in piglets at 26 dpn compared to animals at 5 and 12 dpn in both muscles (*p* < 0.001, over groups). LBW piglets tended to have smaller muscle fibers than NBW piglets within MLD (*p* = 0.062) and within the MST (*p* = 0.088). Moreover, Gln-supplemented piglets had larger muscle fibers within MLD (446.1 µm^2^, *p* = 0.048) than Ala-supplemented animals (410.1 µm^2^). The apparent total muscle fiber number of MLD was higher in 26 dpn compared to 5 dpn piglets (*p* = 0.047, over groups). In the MST, the apparent total muscle fiber number was higher in 26 dpn than in 5 and 12 dpn piglets (*p* < 0.001). LBW piglets had fewer (*p* < 0.01) muscle fibers (MLD: 10.6 × 10^5^; MST: 7.0 × 10^5^) compared to NBW piglets (MLD: 12.2 × 10^5^; MST: 8.0 × 10^5^) in both muscles. An influence of supplementation on the apparent total muscle fiber number was not detected.

**Figure 2:**
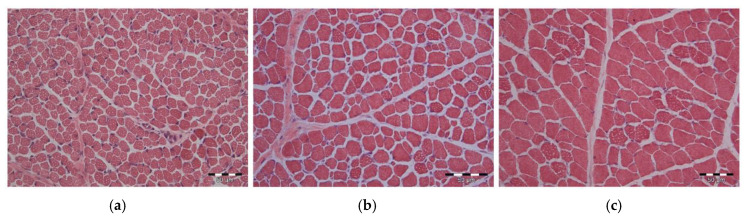
(**a**–**c**): Hematoxylin and eosin (H/E)-stained longissimus muscle cross sections of piglets at 5, 12 and 26 dpn, respectively. Nuclei appear dark blue. Scale bar = 50 µm.

Muscle sections stained with H/E were used to determine the number of nuclei per area unit and per muscle fiber (Figure 2a–c). The nuclei number per mm^2^ (Table 2 and Table 3) decreased with age from 5 dpn to 26 dpn significantly (*p* < 0.001). The muscle fiber size became larger at the same time, thus significant differences in the number of nuclei per muscle fiber were not detected (*p* = 0.105). There was only a trend for fewer myonuclei per muscle fiber within MLD in LBW compared to NBW piglets, independent of supplementation, at 5 (*p* = 0.087) and 26 dpn (*p* = 0.075), and within the MST in LBW piglets at 5 (*p* = 0.08) and 12 dpn (*p* = 0.068). No influence of Gln supplementation was detected (*p* = 0.168).

**Figure 3:**
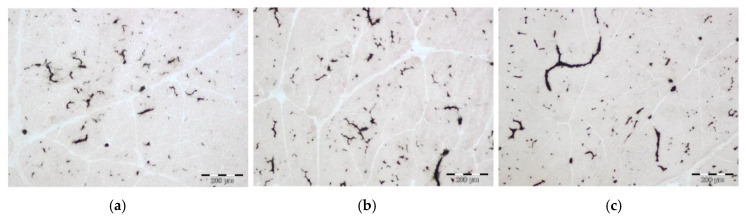
(**a**–**c**): Alkaline phosphatase/eosin stained longissimus muscle cross sections of piglets at 5, 12 and 26 dpn, respectively. Capillaries were stained black. Scale bar = 200 µm.

The capillarization was investigated to assess the potential nutrient and oxygen supply of the muscle by circulation and to detect possible effects of BiW and AA supplementation (Figure 3, Table 2 and Table 3). The results indicated a trend for fewer capillaries (*p* = 0.085) in MLD of LBW piglets (113.2 per mm^2^) compared to NBW piglets (122.3 per mm^2^) over ages, independent of supplementation. Capillary density was lower in piglets at 5 dpn than in piglets at 12 (*p* = 0.004) and 26 dpn (*p* = 0.003). Capillary size in MLD was smaller (*p* = 0.04) at 12 dpn in Gln-supplemented compared to Ala-supplemented piglets (Table 2). Capillary size was greater in piglets at 12 dpn compared to 5 dpn (*p* < 0.001) and 26 dpn (*p* = 0.001), over ages, independent of supplementation. The capillary density in the MST was lower in NBW-GLN piglets compared to NBW-ALA piglets at 26 dpn (*p* = 0.028) and was not affected by age (*p* = 0.879). Capillary size was larger (*p* < 0.001) in piglets at 5 and 12 dpn compared to 26 dpn.

### 3.4. Abundance of MYH Isoforms

**Figure 4:**
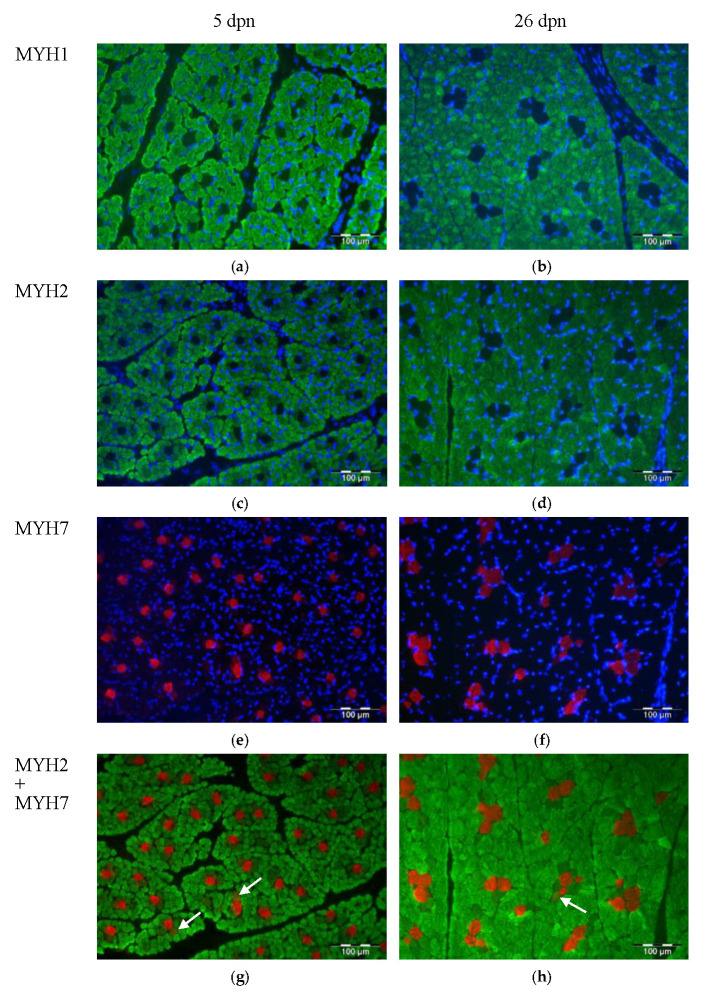
Localization of myosin heavy chain (MYH) isoforms within M. longissimus of piglets at 5 (**a**,**c**,**e**,**g**) and 26 dpn (**b**,**d**,**f**,**h**). MYH1 (**a**,**b**) and MYH2 (**c**,**d**,**g**,**h**) were stained green; MYH7 (**e**–**h**) was stained red. Nuclei were counterstained with Hoechst 33258 and appear in blue. Arrows in MYH2 and MYH7 double staining images (**g**,**h**) indicate transient fibers. Scale bar = 100 µm.

Myosin heavy chain isoforms were localized in muscle sections with immunohistochemistry, as shown in Figure 4. Fast MYH isoforms (MYH1 and 2) were predominant in MLD in 5 dpn and 26 dpn piglets (Figure 4a–d), while slow myosin was less abundant and usually observed in different muscle fibers (Figure 4e,f). Fibers expressing MYH2 and MYH7 at the same time were also detected (arrows in Figure 4g,h), suggesting transition from one fiber type to the other. The antibody against MYH4 did not show a specific staining pattern with immunohistochemistry, while it worked well in western blot.

Results of protein abundance of MYH isoforms are shown as LSmeans and SE for the BiW × Sup × Age interaction, BiW × Sup over ages, and for Age over groups in Table 4 and Table 5 for the MLD and MST, respectively. For MYH1 and MYH4 abundance in MLD, no influence of the single effects BiW (*p* = 0.476, *p* = 0.333), Gln supplementation (*p* = 0.769, *p* = 0.895) or age (*p* = 0.552, *p* = 0.392) was detected. However, there was a trend for more MYH1 protein in LBW piglets at 12 dpn (*p* = 0.074). The protein abundance of MYH2 was lower in MLD of piglets at 26 dpn in comparison with piglets at 5 dpn (*p* = 0.026) and 12 dpn (*p* = 0.022). Compared to Ala-supplemented piglets, the protein abundance of MYH2 tended to be higher in Gln-supplemented piglets at 12 dpn (*p* = 0.055), but this trend was not observed in piglets at 5 dpn (*p* = 0.984) or 26 dpn (*p* = 0.882). In contrast, the MYH7 relative protein abundance in the MLD was not different between 26 dpn and 5 dpn (*p* = 0.228), and tended to be lower in Gln-supplemented piglets at 5 dpn (*p* = 0.065). Similar results were obtained for the MST (Table 5). No clear indications of BiW (*p* = 0.709), supplementation (*p* = 0.949) or age (*p* = 0.071) effects were found for MYH1 relative protein abundance in the MST. Furthermore, MYH2 protein abundance was lower in piglets at 26 dpn compared to piglets at 12 dpn (*p* = 0.045) and 5 dpn (*p* = 0.062), but it was not influenced by BiW (*p* = 0.207) or Gln supplementation (*p* = 0.612). The MYH7 protein abundance was lower in NBW-GLN piglets compared to NBW-ALA piglets at 26 dpn (*p* = 0.006) and was lower in LBW-ALA than in NBW-ALA piglets (*p* < 0.001). MYH4 protein was not determined in the MST because of the unspecific staining pattern in MLD and the absence of differences among groups in western blot results of MLD.

### 3.5. Intramuscular Lipid Deposition

**Figure 5:**
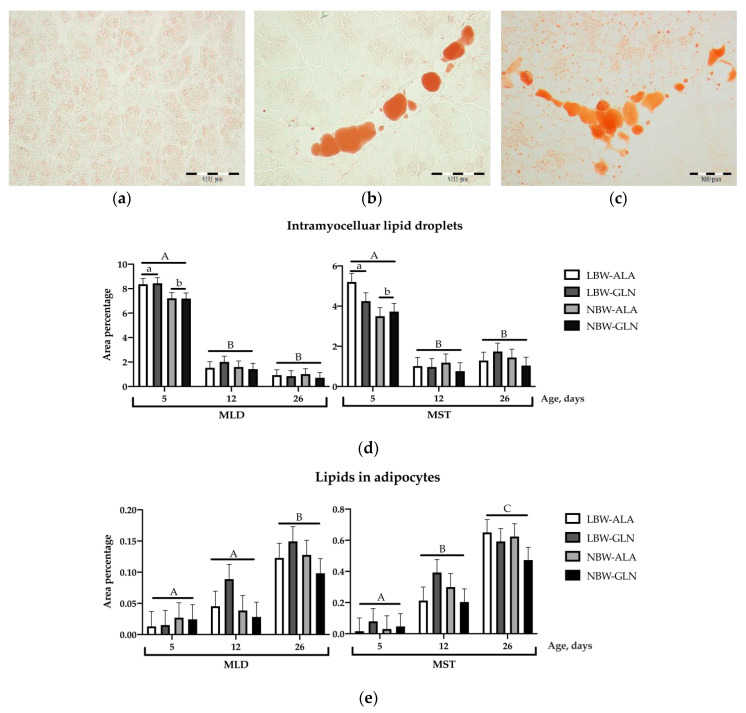
Intramuscular lipid deposition in M. longissimus (MLD) and M. semitendinosus (MST) of low (LBW) and normal birth weight piglets (NBW) supplemented with glutamine (GLN) or alanine (ALA) between 1 and 12 dpn (*n* = 12 per group and age). (**a**–**c**): Oil Red O-stained longissimus muscle sections of piglets at 5, 12 and 26 dpn, lipids are stained red. Scale bar = 100 µm. (**d**): Relative area of intramyocellular lipid droplets. (**e**): Relative area of intramuscular lipids in adipocytes. Values are LSmeans with SE in error bars; ^a,b^ indicate significant differences among groups at the same age; ^A–C^ indicate significant differences among ages (*p* ≤ 0.05, Tukey–Kramer test).

Intramuscular lipid deposition was quantified using Oil Red O-stained muscle slices. Two categories were separately analyzed, namely intramyocellular lipid droplets and lipids in developing adipocytes. Examples of stained muscle sections of MLD at 5, 12 and 26 dpn are shown in Figure 5a–c. Intramyocellular lipid droplets decreased massively from 5 dpn to 12 dpn in both muscles (*p* < 0.001) and stayed unchanged thereafter until 26 dpn (*p* = 0.319 and *p* = 0.51, for MLD and MST, respectively, Figure 5d). Furthermore, LBW piglets had more lipid droplets within muscle fibers compared to NBW piglets at 5 dpn in both muscles (*p* < 0.001 and *p* = 0.032). For lipid droplets in developing adipocytes, greater area percentage was observed in piglets at 26 dpn compared to younger piglets at 5 or 12 dpn in both muscles (*p* ≤ 0.001, Figure 5e). Supplementation had no effect on lipid deposition (*p* = 0.72 and *p* = 0.91, for MLD and MST, respectively).

## 4. Discussion

The dietary supplementation of AAs to pigs has received increasing interest in recent years [9,15]. For instance, arginine supplementation was shown to promote skeletal muscle growth, immunity and feed efficiency in young pigs [32,33,34] and supplemented branched chain AAs (Leu, Ile and Val) stimulated protein synthesis in skeletal muscle [35], while diets with Pro addition improved body growth of piglets [15]. Growth improvement has been achieved in some studies of dietary Gln supplementation in weaned piglets, but not in neonatal piglets, as reviewed by Wu et al. [10]. In weaned piglets with IUGR, Gln supplementation increased body weight gain and enhanced immune response [36]. The pigs in that study received a dosage of 1 g Gln/kg body weight twice a day, which is twofold higher than the dosage in our study with newborn piglets. Dosage and the developmental stage of the animals at which they receive the supplementation are obviously important factors for the achieved effects. In other species such as mice, Gln supplementation failed to promote weight gain [37]. Nevertheless, most of the studies focused on the intestine development under the influence of Gln supplementation, while skeletal muscle as the main site for Gln synthesis and storage in the body raised little attention [10]. Thus, the aim of the current study was to elucidate the effects of dietary Gln supplementation as well as BiW differences on skeletal muscle morphology of piglets during the early postnatal period. The sows in the experiment were all of the same parity (gilts), fed the same rations and raised under identical production conditions to reduce the number of influencing factors. Sow was also included in the statistical model as a random effect to account for groups of piglets being raised by the same sow. Additionally, milk composition from the sows was similar to previously published compositions (data not shown). Thus, the obtained results cannot be attributed to the sows’ diet.

### 4.1. Intramuscular Amino Acid Concentrations Were Altered by Oral Gln Supplementation

Intramuscular Gln concentration was shown to be positively related to protein synthesis in rats [38,39] and cultured chicken [40] skeletal muscle. Thus, our hypothesis was that a positive relationship exists between intracellular Gln concentration and skeletal muscle growth in young pigs. In perfused rat skeletal muscle, even though intramuscular Gln concentration was positively correlated with protein synthesis rate, its concentration had a limit of about 30 µmol/g on protein synthesis in the presence of insulin [38]. In our study, a maximum concentration of Gln of 5.2 µmol/g was observed. In previous studies, intracellular Gln concentration increased in jejunum and plasma in weanling piglets [41] and rats [42] by oral Gln supplementation. In addition, intramuscular Gln concentration was increased in 28-day-old weanling piglets supplemented with Gln for 7 days [43]. However, to our knowledge, few studies investigated consequences of the intramuscular Gln availability for skeletal muscle development, especially in pigs. In the present study, intramuscular Gln concentration was altered by Gln supplementation in piglets at 5 dpn, however, this effect was not visible in 12 and 26 dpn piglets. We can only speculate that the ingested Gln was bound in the tissue and was not measurable as free AA. Thus, effects of supplementation were still possible. The cellular Ala and other free AA concentrations within MLD were influenced at a younger age of 5 and 12 dpn as well. Therefore, supplementation had obviously short-term effects on the availability of essential AA in muscle tissue and might have affected muscle growth, particularly in early postnatal piglets, with some persistent or long-term effects. Furthermore, Leu, Ile and Val are components for Gln and Ala synthesis in skeletal muscle and other tissues, as reviewed by Wu et al. [15]. We observed higher Val concentrations in Gln-supplemented piglets at 12 dpn. Although the AAs were slightly influenced by supplementation, many AAs, e.g., Leu, Ile, Val, Asp, Pro, etc., were more abundant in LBW piglets at 5 or 12 dpn, indicating that these AAs were not sufficiently used in LBW for protein synthesis and muscle growth at a young age. The difference between LBW and NBW piglets disappeared at 26 dpn, suggesting that AAs were increasingly required for protein synthesis in LBW piglets. Furthermore, Asp, Val and Pro were reported to be more abundant in oxidative muscles [44,45]. This could be an evidence for more oxidative fibers in LBW piglets, which is discussed below.

### 4.2. Muscle Fiber Size Was Influenced by Gln Supplementation

In skeletal muscle, muscle fiber type, abundance of intramyocelluar lipid droplets, intramuscular adipocytes, nuclei and connective tissue, as well as capillary density, reflect growth performance of pigs and pork quality at the cellular level [46,47,48]. Generally, total muscle fiber number is fixed at birth in pigs, because primary and secondary fibers are formed during gestation [16], and the growth of muscle after birth is mainly determined by muscle fiber hypertrophy [49]. However, tertiary fibers with smaller size were discovered that develop after birth, even though they play a minor role in muscle development [16,50]. In the present study, we found greater apparent total muscle fiber numbers in both the MLD and MST at 26 dpn compared to 5 dpn. The results indicate that there was a potential for increasing the size and number of muscle fibers in the early postnatal phase of piglets that can be supported by dietary supplementation. Consistent with Gondret’s study [51], who reported that LBW piglets had fewer myofibers in the MLD compared to high birth weight piglets, we observed a trend for fewer muscle fibers in both muscles of LBW piglets in comparison with NBW littermates. Moreover, Gondret and colleagues found that LBW piglets had larger muscle fibers at 112 kg of body weight, but in young piglets in our study, muscle fibers tended to be smaller in LBW piglets. This suggests that LBW piglets catch up growth via increasing muscle hypertrophy and fat deposition [6,51]. Notably, the results of our study suggest that Gln supplementation promoted the growth of muscle fibers within the MLD and imply that dietary addition of Gln could stimulate piglets’ skeletal muscle growth in early life. However, whether this greater muscle fiber size was caused by faster protein synthesis or extracellular fluid expansion from Gln supplementation [52] in the muscle tissue needs further investigations.

Capillary distribution can be an indicator of the nutrient and oxygen supply within skeletal muscle. It was hypothesized that capillarization is closely related to oxidative fibers [53,54]. Nonetheless, Maxwell et al. failed to find correlations between capillarization and oxidative capacity in several muscles of cats and other small mammals [55]. Bauer and colleagues [55,56] found that the ratio of type I fibers to total fibers and the capillary density, as well as blood flow within muscle fibers, was greater in the flexor digitalis superficialis and gastrocnemius medialis of 1-day-old IUGR piglets compared to pigs with normal BiW. Additionally, clear correlations between type I fibers and capillarization were observed in flexor muscles [56]. However, in contrast to that study indicating that IUGR piglets have higher muscle blood supply [56,57], we still found a trend of smaller capillary density within the MLD in LBW piglets with no effects of supplementation or age, although our LBW piglets were not IUGR. Particular muscles may be differently affected by low BiW or IUGR. The trend for a lower capillarization of LBW piglets may explain the diminished supplementation effect on Gln concentration in the MLD. Therefore, BiW might be a potential factor influencing capillarization; however, we found no indication that Gln supplementation can modulate the capillary density development.

The nuclei within muscle fibers and associated satellite cells are the basis of muscle growth and regeneration [58] and can indicate the potential for muscle growth. In this study, myonuclei number per area unit declined with age in all groups, consistent with a previous study [59], which could be explained by hypertrophy of muscle fibers with increasing age. This negative relationship of nuclei number and muscle fiber size was also found in other studies [60]. LBW piglets tended to have fewer nuclei within both muscles at different ages, whereas the number of nuclei per muscle fiber remained almost constant until 26 dpn in the present study. This, together with lower apparent total muscle fiber number in LBW piglets, could be an indication for the diminished muscle growth. Supplementation with Gln apparently did not alter the ratio of nuclei number per muscle fiber.

### 4.3. Abundance of MYH Isoforms Was Slightly Altered by Gln Supplementation

The four types of fibers in adult pig skeletal muscle are named type I, IIa, IIb and IIx, which are composed of different myosin heavy chain (MYH) isoforms [48] coded by MYH7, MYH2, MYH4 and MYH1 genes, respectively. Co-expression of different MYH isoforms forms hybrid muscle fibers [61], as was also shown in our study. In pig production, muscle fiber type composition is a factor influencing the meat quality [62]. More slow fibers promote tenderness, juiciness and flavor of pork [51,62]. However, compared to slow oxidative fibers, fast glycolytic fibers have a higher growth potential [49]. Therefore, it is contradictory to develop both body growth and meat quality in pigs at the same time. Our results indicated a trend for decreased MYH7 protein abundance upon Gln supplementation in the MLD of piglets at 5 dpn. Moreover, less MYH7 was measured in Gln-supplemented NBW piglets at 26 dpn within the MST, suggesting muscle fiber conversion from slow to fast type due to the supplementation. There was no strong evidence for this conversion from differences in MYH isoform abundance. However, we observed a higher intramuscular CAR concentration in Gln-supplemented piglets at 12 dpn, suggesting a higher number of fast glycolytic fibers, as CAR is usually enriched in those fibers [45]. Results of previous studies suggested that LBW piglets with IUGR had more slow muscle fibers within the flexor digitalis superficialis and gastrocnemius medialis [63]. Higher MYH7 abundance could have pointed to the same direction in our study, but similar results were not observed in the MLD or MST of LBW piglets, even though higher oxidative fiber-related AA concentrations were observed in these animals. Thus, IUGR could be a factor influencing the muscle fiber types, in contrast to LBW without IUGR. Altogether, these results indicate that Gln supplementation and BiW had only minor effects on MYH protein abundance within skeletal muscle.

### 4.4. Intramyocellular Lipid Droplets Were More Abundant in LBW Piglets

Considering the lipogenesis in early postnatal piglets, lipid development competes with muscle growth [64], thus LBW piglets normally have a higher content of lipids and smaller muscle mass at slaughter age, as reviewed by Rehfeldt et al. [65]. Lipids in muscle are stored either within muscle fibers as intramyocellular lipid droplets or within adipocytes as intramuscular or marbling fat. Shortly after birth, piglets usually have no intramuscular fat [19]. All lipids are stored in intramyocellular lipid droplets, as observed in our study. A higher content of intramyocelluar lipid droplets was observed in LBW piglets compared to their NBW littermates at 5 dpn. Differences between LBW and NBW piglets disappeared with developing adipocytes, as seen from 12 dpn onwards. However, there was no difference of intramuscular lipid content in adipocytes between LBW and NBW piglets in our study. The fat within skeletal muscle was not fully developed at these young ages, as also described in other reports [50]. An effect of Gln supplementation on lipid deposition within the MLD and MST was not observed. However, the obvious conversion of lipids within muscle tissue from intramyocellular droplets to adipocytes with increasing age and underlying factors, as well as potential modulators, might be an interesting topic for follow-up studies.

## 5. Conclusions

The current study confirmed that BiW influenced the skeletal muscle growth of neonatal piglets, as shown for muscle fiber size and apparent total muscle fiber number, as well as intramuscular lipid development. However, the Gln supplementation had some minor effects on modulating the muscle morphology during the investigated early postnatal period. Whether this is related to the used level of supplemented Gln cannot be determined from the present data. Nevertheless, potential Gln-associated changes in gene expression and cellular differentiation events warrant further investigation.

## Figures and Tables

**Table 1:** Concentrations of intramuscular free amino acids and their metabolites (µmol/kg fresh weight) within M. longissimus of low (LBW) and normal birth weight (NBW) piglets supplemented with glutamine (GLN) or alanine (ALA) between 1 and 12 dpn (*n* = 12 per group and age).

Item	Age	LBW-	LBW-	NBW-	NBW-	SE	Age	SE	*p*-Value		
		ALA	GLN	ALA	GLN				BiW	Sup	BiW × Sup
Arg	5	126	144	134	176	47	145 ^B^	23	0.670	0.520	0.882
	12	534	468	414	526	47	495 ^A^	23	0.282	0.950	0.089
	26	131	160	134	92	47	129 ^B^	23	0.493	0.888	0.782
Leu	5	153	146	133	129	12	140	8	0.057	0.634	0.269
	12	154 ^a^	153 ^a^	114 ^b^	129 ^a^^,^^b^	13	138	9	0.001	0.538	0.007
	26	146	153	143	143	12	146	8	0.501	0.727	0.866
Ile	5	138	137	124	108	10	127 ^A^	6	0.028	0.401	0.107
	12	82	99	63	71	11	79 ^B^	6	0.017	0.228	0.061
	26	113	99	96	97	10	101 ^A^	6	0.298	0.505	0.548
Lys	5	241	291	180	210	51	231 ^B^	39	0.043	0.343	0.163
	12	299	264	254	251	53	267 ^B^	41	0.409	0.664	0.780
	26	444 ^a,b^	530 ^a^	396 ^a,b^	373 ^b^	49	436 ^A^	36	0.004	0.490	0.010
Val	5	221 ^a^	207 ^a,b^	182 ^b^	182 ^a,b^	14	198	10	0.003	0.574	0.020
	12	205 ^a^	226 ^a^	159 ^b^	192 ^a,b^	13	195	10	<.001	0.033	<.001
	26	181	16 7	175	171	14	173	9	0.904	0.486	0.862
Gln	5	5198 ^a,b^	6177 ^a^	4665 ^b^	5945 ^a^	357	5496 ^A^	222	0.211	0.001	0.007
	12	3682	4321	4006	4336	365	4086 ^B^	229	0.578	0.166	0.477
	26	3393	2727	3047	3085	348	3063 ^C^	210	0.985	0.373	0.545
Ala	5	9453 ^a^	7360 ^b^	8738 ^a^	6935 ^b^	357	8122 ^A^	209	0.078	<.001	<.001
	12	6436 ^a^	5075 ^b^	6402 ^a^	4885 ^b^	362	5700 ^B^	213	0.728	<.001	0.001
	26	3427	3533	3360	3499	350	3455 ^C^	199	0.876	0.730	0.986
Glu	5	3934	4175	3908	3500	225	3879 ^A^	140	0.070	0.700	0.101
	12	3678	3291	3523	3055	230	3387 ^B^	145	0.310	0.053	0.187
	26	2204	2227	2222	2047	219	2175 ^C^	133	0.675	0.732	0.904
Asp	5	702	663	619	588	384	643 ^A^	23	0.022	0.347	0.101
	12	511	518	497	417	391	486 ^B^	24	0.094	0.335	0.149
	26	316	365	327	349	75	339 ^C^	22	0.938	0.349	0.789
Cit	5	252	288	239	267	20	261 ^A^	14	0.246	0.069	0.194
	12	293	323	29 6	292	21	301 ^A^	15	0.343	0.456	0.424
	26	153	146	138	120	19	139 ^B^	13	0.173	0.506	0.486
Orn	5	142	175	173	150	38	160 ^B^	24	0.931	0.891	0.855
	12	322 ^a,b^	364 ^a^	316 ^a,b^	233 ^b^	39	309 ^A^	25	0.034	0.574	0.036
	26	154	190	134	99	37	144 ^B^	23	0.085	0.987	0.244
Pro	5	4766 ^a,b^	5164 ^a^	4132 ^a,b^	4099 ^b^	395	4541 ^A^	293	0.003	0.588	0.019
	12	1840	2373	2165	1822	410	2162 ^B^	309	0.686	0.704	0.113
	26	1815	1804	1567	1798	380	1746 ^B^	273	0.645	0.761	0.918
Gly	5	7347	7002	8123	7486	365	7490 ^B^	244	0.032	0.146	0.074
	12	9537	9979	10284	10399	376	10080 ^A^	254	0.047	0.419	0.176
	26	7713	8161	7722	7458	354	7764 ^B^	229	0.234	0.795	0.394
β-Ala	5	3051	3232	2585	2601	258	2867 ^A^	182	0.005	0.667	0.041
	12	1730	1685	1480	1181	267	1519 ^B^	191	0.053	0.462	0.191
	26	863	531	762	552	249	677 ^C^	170	0.835	0.267	0.709
Car	5	2774	3348	3093	3551	455	3192 ^C^	268	0.519	0.245	0.615
	12	8633	9615	8159	9637	462	9011 ^B^	275	0.577	0.007	0.050
	26	13738	14178	13818	13613	445	13837 ^A^	256	0.549	0.795	0.785
Anser	5	48	55	53	62	10	54 ^C^	8	0.362	0.313	0.592
	12	129 ^b^	141 ^a,b^	141 ^a,b^	159 ^a^	10	142 ^B^	8	0.019	0.060	0.028
	26	268 ^b^	282 ^a,b^	296 ^a^	297 ^a^	9	286 ^A^	7	0.001	0.348	0.005

BiW: birth weight; Sup: supplement; Arg: arginine; Leu: leucine; Ile: isoleucine; Lys: lysine; Val: valine; Gln: glutamine; Ala: alanine; Glu: glutamic acid; Asp: aspartic acid; Cit: citrulline; Orn: ornithine; Pro: proline; Gly: glycine; β-Ala: beta-alanine; Car: carnosine; Anser: anserine. Values represent least square means (LSmeans) and standard errors (SE), the largest SE is shown; ^a,b^ indicate significant differences among groups at the same age; ^A–C^ indicate significant differences among ages (*p* ≤ 0.05, Tukey–Kramer test).

**Table 2:** Morphological traits in M. longissimus of low (LBW) and normal birth weight (NBW) piglets supplemented with glutamine (GLN) or alanine (ALA) between 1 and 12 dpn (*n* = 12 per group and age).

Trait	Age	LBW-	LBW-	NBW-	NBW-	SE	Age	SE	*p*-Value		
		ALA	GLN	ALA	GLN				BiW	Sup	BiW × Sup
Muscle cross sectional area (mm^2^)	5	238.5	250.7	300.3	327.1	33.1	279.1 ^C^	24.4	0.004	0.492	0.030
	12	404.6 ^b^	455.9 ^a,b^	497.2 ^a^	510.1 ^a^	34.4	467.0 ^B^	25.7	0.002	0.268	0.011
	26	635.4 ^b^	668.2 ^b^	791.8 ^a^	784.6 ^a^	32.0	720.0 ^A^	22.7	<0.001	0.675	<0.001
	Overall	426.1 ^b^	458.3 ^b^	529.8 ^a^	540.6 ^a^	19.2					
Muscle fiber size (µm^2^)	5	257.1	280.0	271.0	312.4	32.5	280.1 ^C^	20.8	0.393	0.297	0.584
	12	393.8	391.1	405.2	401.9	33.4	398.0 ^B^	21.5	0.682	0.924	0.981
	26	538.5 ^b^	619.6 ^a,b^	594.8 ^a,b^	671.8 ^a^	31.7	606.2 ^A^	19.6	0.048	0.014	0.021
	Overall	396.5 ^b^	430.3 ^a,b^	423.7 ^a,b^	462.0 ^a^	18.8					
Apparent total muscle fiber number (×10^5^)	5	8.9	9.6	10.9	11.4	0.9	10.2 ^B^	0.6	0.022	0.562	0.129
	12	10.5	12.2	12.3	12.8	0.9	11.9 ^A,B^	0.6	0.129	0.231	0.230
	26	11.7	10.8	14.0	11.9	0.9	12.1 ^A^	0.5	0.042	0.100	0.063
	Overall	10.4	10.8	12.4	12.0	0.5					
Number of nuclei per muscle fiber	5	0.61	0.63	0.69	0.70	0.05	0.66	0.03	0.087	0.814	0.384
	12	0.61	0.64	0.62	0.58	0.05	0.61	0.03	0.620	0.864	0.865
	26	0.62	0.69	0.73	0.74	0.05	0.70	0.03	0.075	0.422	0.239
	Overall	0.62	0.65	0.68	0.67	0.03					
Capillary density (number per mm^2^)	5	84.1	88.8	98.9	100.3	11.3	93.0 ^B^	7.5	0.149	0.772	0.527
	12	113.7	137.5	122.6	147.4	11.7	130.3 ^A^	7.9	0.300	0.025	0.108
	26	133.7	121.5	140.8	123.8	11.0	130.0 ^A^	7.1	0.602	0.187	0.149
	Overall	110.5	115.9	120.7	123.8	6.5					
Capillary size (µm^2^)	5	81.2	87.6	86.5	99.3	9.5	88.7 ^B^	5.9	0.298	0.290	0.500
	12	143.7	121.9	140.2	123.5	9.8	132.3 ^A^	6.1	0.904	0.040	0.228
	26	100.6	113.6	93.3	96.0	9.3	100.9 ^B^	5.6	0.129	0.404	0.334
	Overall	108.5	107.7	106.7	106.3	5.5					

BiW: birth weight; Sup: supplementation. Values represent LSmeans and standard errors (SE), the largest SE is shown; ^a,b^ indicate significant differences among groups at the same age; ^A–C^ indicate significant differences among ages (*p* ≤ 0.05, Tukey–Kramer test).

**Table 3:** Morphological traits in M. semitendinosus of low (LBW) and normal birth weight (NBW) piglets supplemented with glutamine (GLN) or alanine (ALA) between 1 and 12 dpn (*n* = 12 per group and age).

Trait	Age	LBW-	LBW-	NBW-	NBW-	SE	Age	SE	*p*-Value		
		ALA	GLN	ALA	GLN				BiW	Sup	BiW × Sup
Muscle cross sectional area (mm^2^)	5	302.7	275.5	307.1	279.3	54.7	291.1 ^B^	44.3	0.900	0.509	0.928
	12	395.0	400.0	389.4	460.3	57.3	411.1 ^B^	46.9	0.407	0.369	0.476
	26	605.3	543.8	644.1	659.3	52.3	613.1 ^A^	40.9	0.022	0.613	0.076
	Overall	434.3	406.3	446.9	466.3	31.7					
Muscle fiber size (µm^2^)	5	375.6	407.1	434.2	460.5	42.6	419.4 ^C^	25.2	0.142	0.486	0.447
	12	541.7	576.0	563.3	594.5	43.4	568.9 ^B^	25.8	0.598	0.439	0.830
	26	828.6	865.7	900.3	867.8	41.7	865.6 ^A^	24.1	0.332	0.957	0.617
	Overall	582.0	616.3	632.6	640.9	24.6					
Apparent total muscle fiber number (×10^5^)	5	0.39	0.41	0.53	0.57	0.09	0.47 ^B^	0.07	0.012	0.661	0.084
	12	0.61	0.61	0.74	0.70	0.09	0.66 ^B^	0.07	0.061	0.794	0.290
	26	1.20	0.98	1.17	1.13	0.08	1.12 ^A^	0.06	0.338	0.096	0.115
	Overall	0.73 ^a,b^	0.67 ^b^	0.81 ^a^	0.80 ^a^	0.05					
Number of nuclei per muscle fiber	5	0.55	0.54	0.56	0.71	0.05	0.59 ^B^	0.03	0.080	0.205	0.080
	12	0.60	0.63	0.62	0.79	0.05	0.66 ^B^	0.03	0.068	0.068	0.043
	26	0.80	0.79	0.85	0.78	0.05	0.81 ^A^	0.03	0.719	0.474	0.823
	Overall	0.65	0.65	0.68	0.76	0.03					
Capillary density (number per mm^2^)	5	82.2	75.5	82.9	77.6	8.3	79.6	4.1	0.869	0.468	0.905
	12	80.7	77.3	76.7	76.2	8.3	77.7	4.1	0.762	0.814	0.981
	26	73.2 ^a,b^	84.7 ^a,b^	98.9 ^a^	65.8 ^b^	8.3	80.6	4.1	0.684	0.197	0.031
	Overall	78.7	79.2	86.2	73.2	4.8					
Capillary size (µm^2^)	5	200.3	211.8	230.9	225.1	17.8	217.0 ^A^	10.3	0.177	0.869	0.543
	12	199.1	188.6	172.8	205.5	18.1	191.5 ^A^	10.5	0.774	0.534	0.523
	26	135.8	109.2	114.8	128.2	17.5	122.0 ^B^	9.8	0.948	0.711	0.646
	Overall	178.4	169.9	172.8	186.3	10.3					

BiW: birth weight; Sup: supplementation. Values represent LSmeans and standard errors (SE), the largest SE is shown; ^a,b^ indicate significant differences among groups at the same age; ^A–C^ indicate significant differences among ages (*p* ≤ 0.05, Tukey–Kramer test).

**Table 4:** Relative normalized protein abundance of MYH isoforms in M. longissimus of low (LBW) and normal birth weight (NBW) piglets supplemented with glutamine (GLN) or alanine (ALA) between 1 and 12 dpn (*n* = 12 per group and age).

Item	Age	LBW-	LBW-	NBW-	NBW-	SE	Age	SE	*p*-Value		
		ALA	GLN	ALA	GLN				BiW	Sup	BiW × Sup
MYH1	5	0.49	0.48	0.52	0.51	0.07	0.50	0.05	0.643	0.855	0.968
	12	0.64	0.59	0.46	0.57	0.07	0.57	0.05	0.074	0.599	0.140
	26	0.42	0.58	0.57	0.44	0.07	0.50	0.04	0.935	0.862	0.115
	Overall	0.52	0.55	0.52	0.51	0.04					
MYH2	5	0.51	0.51	0.53	0.54	0.06	0.52 ^A^	0.04	0.619	0.984	0.969
	12	0.49	0.52	0.47	0.64	0.06	0.53 ^A^	0.04	0.210	0.055	0.051
	26	0.35	0.36	0.42	0.39	0.06	0.38 ^B^	0.04	0.240	0.882	0.662
	Overall	0.45	0.46	0.48	0.52	0.03					
MYH4	5	0.31	0.35	0.23	0.30	0.05	0.30	0.03	0.144	0.305	0.360
	12	0.29	0.23	0.23	0.22	0.05	0.24	0.03	0.411	0.487	0.698
	26	0.24	0.27	0.32	0.24	0.05	0.27	0.03	0.531	0.555	0.581
	Overall	0.28	0.28	0.26	0.25	0.03					
MYH7	5	20.31	12.73	13.95	13.02	2.58	15.00	1.83	0.117	0.065	0.035
	12	9.44	14.26	13.96	11.60	2.62	12.32	1.92	0.628	0.579	0.262
	26	8.57	10.18	10.27	13.53	2.51	10.64	1.71	0.211	0.310	0.421
	Overall	12.77	12.39	12.73	12.72	1.49					

MYH: myosin heavy chain; BiW: birth weight; Sup: supplementation. Values represent LSmeans and standard errors (SE), the largest SE is shown; ^A,B^ indicate significant differences among ages (*p* ≤ 0.05, Tukey–Kramer test).

**Table 5:** Relative normalized protein abundance of MYH isoforms in M. semitendinosus of low (LBW) and normal birth weight (NBW) piglets supplemented with glutamine (GLN) or alanine (ALA) between 1 and 12 dpn (*n* = 12 for MYH7 and *n* = 6 for MYH1 and 2 per group and age).

Item	Age	LBW-	LBW-	NBW-	NBW-	SE	Age	SE	*p*-Value		
		ALA	GLN	ALA	GLN				BiW	Sup	BiW × Sup
MYH1	5	0.39	0.38	0.33	0.40	0.05	0.38	0.04	0.529	0.511	0.533
	12	0.34	0.33	0.32	0.33	0.05	0.33	0.04	0.800	0.996	0.953
	26	0.42	0.42	0.51	0.44	0.05	0.45	0.03	0.128	0.517	0.250
	Overall	0.38	0.38	0.39	0.39	0.03					
MYH2	5	0.040	0.038	0.040	0.043	0.003	0.040 ^A,B^	0.002	0.177	0.782	0.319
	12	0.042	0.038	0.038	0.041	0.002	0.040 ^B^	0.002	0.898	0.770	0.193
	26	0.046	0.045	0.045	0.049	0.002	0.046 ^A^	0.002	0.340	0.367	0.289
	Overall	0.042	0.040	0.041	0.044	0.001					
MYH7	5	1.29	2.52	0.83	3.30	1.22	1.98	0.95	0.842	0.062	0.248
	12	1.11	1.27	0.92	1.54	1.27	1.21	1.00	0.962	0.696	0.971
	26	2.40 ^b^	3.69 ^a,b^	7.07 ^a^	3.52 ^b^	1.17	4.17	0.88	0.006	0.294	<0.001
	Overall	1.60	2.50	2.94	2.79	0.70					

MYH: myosin heavy chain; BiW: birth weight; Sup: supplementation. Values represent LSmeans and standard errors (SE), the largest SE is shown; ^a,b^ indicate significant differences among groups at the same age; ^A,B^ indicate significant differences among ages (*p* ≤ 0.05, Tukey–Kramer test).
